# Sporotrichosis In Immunocompromised Hosts

**DOI:** 10.3390/jof5010008

**Published:** 2019-01-11

**Authors:** Flavio Queiroz-Telles, Renata Buccheri, Gil Benard

**Affiliations:** 1 Department of Public Health, Federal University of Paraná, Curitiba 80060-000, Brazil; 2 Emilio Ribas Institute of Infectious Diseases, São Paulo 05411-000, Brazil; renatabuccheri@gmail.com; 3 Laboratory of Medical Mycology, Department of Dermatology, and Tropical Medicine Institute, University of São Paulo, Sao Paulo 05403-000, Brazil; bengil60@gmail.com

**Keywords:** AIDS, IRIS, cat-transmitted sporotrichosis, immunocompromised hosts, mycoses of implantation, sporotrichosis, *Sporothrix brasiliensis*, *Sporothrix schenckii*, subcutaneous mycoses

## Abstract

Sporotrichosis is a global implantation or subcutaneous mycosis caused by several members of the genus *Sporothrix*, a thermo-dimorphic fungus. This disease may also depict an endemic profile, especially in tropical to subtropical zones around the world. Interestingly, sporotrichosis is an anthropozoonotic disease that may be transmitted to humans by plants or by animals, especially cats. It may be associated with rather isolated or clustered cases but also with outbreaks in different periods and geographic regions. Usually, sporotrichosis affects immunocompetent hosts, presenting a chronic to subacute evolution course. Less frequently, sporotrichosis may be acquired by inhalation, leading to disseminated clinical forms. Both modes of infection may occur in immunocompromised patients, especially associated with human immunodeficiency virus (HIV) infection, but also diabetes mellitus, chronic alcoholism, steroids, anti-TNF treatment, hematologic cancer and transplanted patients. Similar to other endemic mycoses caused by dimorphic fungi, sporotrichosis in immunocompromised hosts may be associated with rather more severe clinical courses, larger fungal burden and longer periods of systemic antifungal therapy. A prolonged outbreak of cat-transmitted sporotrichosis is in progress in Brazil and potentially crossing the border to neighboring countries. This huge outbreak involves thousands of human and cats, including immunocompromised subjects affected by HIV and FIV (feline immunodeficiency virus), respectively. We reviewed the main epidemiologic, clinical, diagnostic and therapeutic aspects of sporotrichosis in immunocompromised hosts.

## 1. Introduction

Sporotrichosis is a subacute to chronic fungal infection caused by several species of genus *Sporothrix*, a group of thermal dimorphic fungi. Although disease occurs worldwide, most cases are reported in tropical and subtropical zones from Latin America, Africa and Asia [1,2]. Usually, sporotrichosis is an implantation mycosis whose infectious propagules are inoculated from several environmental sources into skin, mucosal or osteoarticular sites [3]. Less frequently, infection may occur by inhalation, resulting in pulmonary disease [4], but in both modalities, immunocompetent and immunocompromised patients can be affected.

In endemic regions, this disease is mainly associated with plant transmission (sapronosis), the main etiologic agents being *S. schenckii* and *S. globosa* [5]. During the last three decades, a new, probable mutant species, *S. brasiliensis*, has emerged in the state of Rio de Janeiro, Brazil. This species is transmitted to humans by infected cats (zoonosis), causing the largest outbreak of sporotrichosis ever reported [6,7,8]. This epizootic outbreak continues to expand, affecting human and feline patients in several Brazilian regions, possibly reaching neighboring countries [9]. Feline sporotrichosis is unique among infections caused by endemic dimorphic fungi because it is directly transmitted in the yeast phase. The feline lesions typically harbor a high yeast-like fungal burden that can be acquired via cat scratches and bites, by non-traumatic ways, such as a cat’s cough or sneezing, and direct contact between patients’ integumental barriers and animal secretions [8].

Similar to other endemic mycoses, sporotrichosis in immunocompromised hosts is usually clinically remarkable. The increased clinical severity is related to a decrease of host immune and inflammatory responses, heavy fungal burden, extensive dissemination and higher mortality rates. In addition, in opportunistic sporotrichosis (OS), conventional serology may reveal false negative antibodies levels and long courses of systemic antifungal therapy are usually required.

This mycosis can affect anyone regardless of age, gender or comorbidities, mostly depending on exposure [1]. Human immunodeficiency virus (HIV)/AIDS changes the natural history of sporotrichosis and its opportunistic character depends on the immune status of the host. Comorbidities such as diabetes mellitus, chronic alcoholism, steroid treatment, hematologic cancer and organ transplantation have been sporadically described as risk factors for severe forms of the disease and case reports have focused on unusual manifestations in these scenarios. The aim of this review is to discuss the main epidemiological, clinical, diagnostic and therapeutic aspects of OS, with an emphasis on cat-transmitted sporotrichosis (CTS). In addition, in an attempt to better understand why certain comorbidities may predispose to OS, we performed a critical review of the data on the immune response in sporotrichosis. 

## 2. Epidemiology and Clinical Manifestations

Sapronotic sporotrichosis is mainly related to several types of transcutaneous injuries, occurring in patients in contact with plant material or contaminated soil. Less frequently, animal associated trauma has been associated with *S. schenckii* and *S. globosa* and, to a lesser extent, with *S. pallida* clade (*S. mexicana*, *S. chilensis, S. luriei* and *S. pallida)* [5,10]. Zoonotic sporotrichosis is caused by *S. brasiliensis*, and although this expanding and uncontrolled outbreak is apparently limited to Brazil’s borders, proven cases have been reported in Argentina and possibly Panama [9,11]. 

Sporotrichosis is a spectral disease, classified into two categories (cutaneous and extracutaneous sporotrichosis), which comprise four distinct clinical forms: lymphocutaneous (LC), fixed cutaneous (FC), disseminated cutaneous, and extra-cutaneous [12]. LC and FC forms are classical and most common clinical presentations [13,14]. Typically, but not exclusively, disseminated cutaneous and extra-cutaneous, considered severe forms, occur in hosts with depressed cellular immunity [15,16,17,18,19]. Indeed, findings from studies that represent the largest reported outbreaks of this mycosis in regions such as China, Japan, Peru and Brazil indicate the frequency of these severe forms ranges from 1.3% to 9% [13,14,20,21].

The disseminated cutaneous form is a rare variant of sporotrichosis characterized by multiple skin lesions at noncontiguous sites without extracutaneous involvement. It is important to emphasize that in some situations it is difficult to identify whether the clinical presentation is due to dissemination from a single lesion or to multiple inoculations [22]. The extracutaneous or disseminated forms are characterized by the involvement of organs and systems. Skin, eyes, lungs, liver, kidney, heart, central nervous system (CNS) and genitalia have already been described as affected sites. The osteoarticular form may occur by contiguity of the primary lesion or hematogenous spread dissemination from lungs [4,23,24,25,26].

Poorly explored until recently, the reemergence of this mycosis in different parts of the world led to a renewed interest in its study, mainly focusing on immunopathogenesis mechanisms and immune response against fungal molecular components; these topics have been reviewed recently [10,27,28]. Nevertheless, our knowledge on the immunopathogenesis of sporotrichosis is still fragmentary.

## 3. What We (Don´t) Know about the Immune Response in Human Sporotrichosis

In sporotrichosis, exposure does not necessarily result in overt disease since the proportion of those who will develop an illness is smaller than those who control the infection. Although the mechanisms underlying this observation are unknown, some studies suggest that different *Sporothrix* species may present different pathogenicity levels, which may lead to varying degrees in clinical manifestations, with some “susceptible” individuals developing the more benign FC form (which can eventually heal spontaneously), while others evolving to severe disseminated or extracutaneous forms.

Current available data on the immune response to *Sporothrix* spp. (or their components) is predominantly based on in vitro studies and in rodent experimental models, reprising the strategies used in the investigation of the immune reactivity of the other, better studied, endemic deep or subcutaneous mycoses; data gathered directly from human patients is scarce. While those studies differ widely in the species tested, the fungal phase used to infect or to obtain fungal components (conidia vs. yeast vs. germlings), the size and route of inoculation (mostly intraperitoneal and intravenous) and animal models employed (mouse strains, Wistar rats, golden hamster and, more recently, the great wax moth *Galleria mellonella*), the extent to which they contribute to the understanding of the immunopathogenesis of the human disease is still not clear since the pieces do not fit the puzzle. Only recently, mouse models mimicking the human disease (i.e., subcutaneous inoculation) have been explored [29,30,31,32].

As a first line of defense against pathogens, innate immunity is considered key to fungal control. Pioneering work by Kajiwara et al. showed that neutrophils and macrophages from mice with chronic granulomatous disease (CGD), who show defective NADPH oxidase complex function and fail to generate microbicidal reactive oxygen species (ROS), were unable to control the growth of *S. schenckii* yeast cells, and those animals developed a disseminated lethal disease upon subcutaneous inoculation, while wild-type counterparts were resistant to systemic infection and survived [32]. Translating those findings, however, to human context seems challenging: while Cunningham et al. observed phagocytosis and intracellular killing of *S. schenckii* by human polymorphonuclear cells in vitro, mediated by the H_2_O_2_–KI–myeloperoxidase system [33], Schafner et al. found that virulent *S. schenckii* was resistant to killing by neutrophils and H_2_O_2_ [34].

The controversial role of nitric oxide (NO) in sporotrichosis highlights the complexity of the host’s immune response in this mycosis. Experimental data suggest a dual role for NO, supporting both its fungicidal activity against *S. schenckii* in vitro [35] and its association with T cell suppression and poorer outcome in murine models [36]. In patients’ biopsies, expression of NO synthase-2 was higher in LC lesions, while FC injuries displayed more intense inflammation, tissue destruction and higher fungal burden [37,38].

Human macrophages were also shown to phagocytose and kill (probably through ROS release) *S. schenckii* conidia and yeast cells [39]. Some studies suggest that melanin expression would protect the isolates from macrophage phagocytosis and oxidative attack [35,40]. However, there are no studies analyzing the in situ expression of melanin by intralesional yeast cells in biopsies. Curiously, in the human monocytic cell line THP-1, engulfment of *S. schenckii* conidia preferentially occurs through mannose receptors while yeast cells internalization relies on complement receptors [39], suggesting the interplay of different receptors in fungi–host interaction.

In parallel to neutrophils and macrophages, it was shown that bone marrow-derived mouse dendritic cells (DC) also participate in the recognition process of fungal components and drive the cellular immune responses [41], regulating the magnitude and balance of Th-1 and Th-17 responses in vitro. The latter were associated with control of the fungal burden in an intraperitoneal infection mouse model [42,43]. Other immune cells, such as mast cells, can also amplify the acute response by releasing mediators (histamine and proinflammatory cytokines that attract neutrophils) that exacerbate the inflammatory process, but with deleterious effects to the host, rather than contributing to control of fungal burden [44,45].

Several studies addressed the recognition of *Sporothrix* spp. and their components by innate immunity receptors (pattern recognition receptors, PRR) and its influence in subsequent cellular immunity. Toll-like receptors (TLR) are conserved membrane-associated proteins that recognize a broad set of microbial components, such as *S. schenckii* lipid antigens, recognized via TLR4 [46], triggering diverse cell responses. TLR2 activation, for example, enhances in vitro phagocytosis of *S. schenckii* yeast cells by mouse macrophages and promotes the release an array of pro- (TNF-α, IL-1β, IL-12) and anti-inflammatory (IL-10) cytokines as well as effector/cytotoxic compounds (e.g., NO) [47,48]. Keratinocytes are also activated through TLR2 and TLR4 to release proinflammatory cytokines when challenged with *S. schenckii* yeast cells [49]. However, it is not yet clear from these studies whether the elicited inflammatory response contributes to enhanced immunopathology or host protection.

Dectin-1 and dectin-2 are important PRRs that trigger Th-17 responses but currently there are only data for participation of dectin-1 in triggering Th-17 responses in an intraperitoneal mouse model of sporotrichosis [50]. Conversely, Zhang et al. showed that both dectin-1 and IL-17 production were dispensable for clearance of *S. schenckii* infection in a rat model [51]. There is also evidence from a mouse model of systemic infection that activation of the inflammasome exerts a transitory protective role, especially due to IL-1, IL-18 and caspase-1 [42,52,53], whose impairment reduced Th-17 and Th-1 mediated inflammatory responses leading to higher susceptibility to *S. schenckii* infection [52]. *S. schenckii* yeast cells can also activate the alternative (antibody independent) complement pathway in vitro but its relevance to in vivo host defenses was not defined [54]. 

Overall, these studies suggest that *Sporothrix* spp. can be recognized by different innate immunity receptors. Which particular set of these (and their signaling pathways) is involved in human infection, which could also be affected by the different infection routes (percutaneous or inhalatory), remains to be determined. Furthermore, with the identification of new *Sporothrix* spp., the involvement of immune receptors could be species-specific.

In fact, Arrillaga-Moncrieff et al. showed that pathogenicity differs among species: *S. brasiliensis* was the most pathogenic, followed by *S. schenckii*, when compared to *S. albicans*, *S. globosa* and *S. mexicana* [55]. Almeida-Paes et al. suggested those differences might even exist within a single species: *S. brasiliensis* isolates obtained from patients with more severe disease express more putative “virulence” factors, such as urease and melanin, and are able to cause a more disseminated disease [56,57]. In Venezuela, a retrospective study gathered isolates from patients and found that *S. globosa* is isolated mainly from patients with FC sporotrichosis while *S. schenckii* would be related to LC forms [58].

Fibronectin surface adhesins expressed by *S. schenckii* have also been described to increase pathogenicity in C57BL/6 mice. Although analyses of differences according to species were not available at that time, they did not find direct correlation between virulence and the clinical or environmental origin of the isolates: the lowest virulence was observed for an isolate recovered from a patient with meningeal sporotrichosis [59].

Recently, Martinez-Alvarez et al. showed that human peripheral blood mononuclear cells (PBMC) differentially recognize *S. brasiliensis* and *S. schenckii* [60]. The three *S. schenckii* morphologies stimulated higher levels of pro-inflammatory cytokines than *S. brasiliensis*, while the latter stimulated higher IL-10 levels. This finding could help to explain the apparent higher pathogenicity of *S. brasiliensis*. However, as we still do not know the first steps of the infection in humans, the contribution of each morphology to its successful occurrence remains to be established. The authors additionally showed that dectin-1 was a key receptor for cytokine production induced by *S. schenckii*, but was dispensable for *S. brasiliensis* germlings. TLR2 and TLR4 were also involved in sensing of *Sporothrix* cells, with a major role for the former during cytokine production. The mannose receptor had a minor contribution in *S. schenckii* yeast-like cells and germlings recognition, but *S. schenckii* conidia and *S. brasiliensis* yeast-like cells stimulated pro-inflammatory cytokines via this receptor. 

Immunochemical studies in the 1970s already suggested that cell wall components elicited immediate and delayed immune responses [61]. Subsequent studies reinforced the important role played by cell-mediated mechanisms (i.e., TCD4+ lymphocytes and activated macrophages) in resistance to intravenous experimental sporotrichosis in athymic nude [62,63,64,65] and Swiss mice [66,67]. This research line was resumed more recently in the search for vaccine candidates. Live yeast cells and/or exoantigens were used, and Th-1 and Th-17 responses were, in general, generated [41,43,53], both of which appeared to be required for protection in these models. However, there was also evidence of an important participation of Th-2 responses at later stages and activation of macrophages with anti-inflammatory characteristics (defined as M2 macrophages) such as high levels of IL-10 secretion [47,48,60,68]. It has been suggested that isolates from cutaneous lesions were more potent to activate human monocyte-derived DCs to drive Th-1 responses than isolates from visceral lesions. However, this finding should be regarded with caution since the study was performed before reclassification of the *S. schenckii* complex into several species and thus the isolates’ differences could rather reflect different species [69].

*Sporothrix* spp. components have also been studied with regard to human humoral responses. Sera from extracutaneous or more severe forms of sporotrichosis recognized a wider range of antigens and displayed higher antibody titers than sera from patients with cutaneous/less severe forms of sporotrichosis [56,70,71]. A protective role of antibodies, possibly through facilitation of phagocytosis, has been described in some experimental models [71,72,73,74]. 

Unfortunately, data stemming from patients are scarce and are represented mostly by histopathology studies of biopsies taken from patients, with a limited set of parameters analyzed due to limitations inherent to these methods. Moreover, some studies involved a rather small number of patients. Nonetheless, the cutaneous inflammatory process consists in most cases of a suppurative granulomatous response, with frequent presence of liquefaction and/or necrosis. Of note, the paucity of fungal elements (absent from 65% of the biopsies), associated with better granuloma formation (epithelioid granulomas, higher infiltration of lymphocytes, presence of fibrosis, absence of necrosis) suggests the ability of the human system in partially containing the fungal burden [75,76]. This may help to explain why (a) exposure does not necessarily result in development of illness and (b) some patients self-heal.

**Figure 1:**
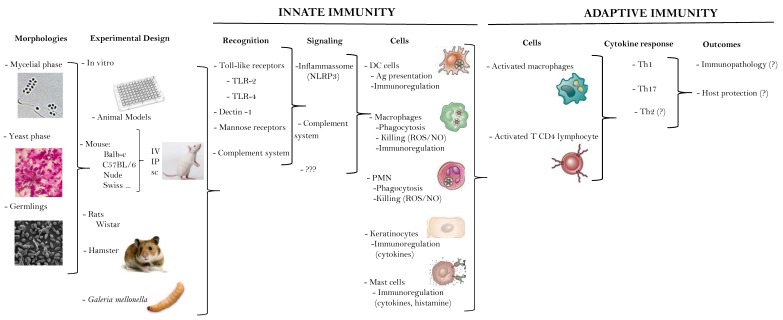
Schematic view of the data obtained from experimental studies of the immune response in sporotrichosis. IV: intravenous; IP: intraperitoneal; sc: subcutaneous; DC: dendritic cells; ROS: reactive oxygen species; NO: nitric oxide; PMN: polymorphonuclear cells; Th: T helper.

Immunohistochemistry studies showed the presence of CD4+ and CD8+ T-cells, CD83+ DC, macrophages and monocytes, and the expression of IFN-γ, but not of iNOS, within granulomas [38,77,78]. Compared to the FC form, LC patients had more intense signs of inflammation (higher infiltration of neutrophils and lymphocytes, and higher expression of nitric oxide synthase 2) and higher fungal burden. IFN-γ expression did not differ but IL-10 was more prominent in LC than FC lesions, consistent with the more intense inflammatory process in the former [77]. Interestingly, these authors also observed a higher ability of PBMC from patients than healthy individuals to release IFN-γand IL-10 upon in vitro challenge with *S. schenckii* antigen [77]. An early report already noted a trend toward lower T-lymphocyte responsiveness in systemic disease as compared with the LC form [78]. Interestingly, of six systemic sporotrichosis patients, one had bone marrow aplasia and four reported daily consumption of variable amounts of alcohol, while none of the LC patients reported these conditions. Overall, these data reinforce the ability of the human immune response in limiting, at least partially, the disease caused by *S. schenckii*. However, this notion can be challenged by the report of severe extracutaneous sporotrichosis in apparently immunocompetent individuals [79,80,81]. A summarized, schematic view of the data obtained from experimental studies of the immune response in sporotrichosis is shown in Figure 1.

## 4. Sporotrichosis in AIDS Patients

The first case report of OS in HIV-infected individuals dates from 1985 [82]. In the last decade, the reemergence of the disease in Rio de Janeiro, Brazil, was followed by an increase in the number of cases in HIV co-infected patients. Nevertheless, to date, there are no more than 107 cases reported worldwide. Reflecting the rare occurrence of the disease in this group, our knowledge of the clinical features and management principles is based on expert opinions, case studies and retrospective cohort studies [12,83,84,85]. Still, it is worth noting that HIV/AIDS patients either had disseminated or cutaneous sporotrichosis, or did not become ill after exposure [12]. 

Data from the largest retrospective cohort study of 3618 cases of sporotrichosis revealed that 1.32% were HIV co-infected. Close to half (44%) were hospitalized over time, much more frequently than HIV negative patients (1%). Although the main cause for hospitalization in both groups was disseminated disease, this corresponded to 90.5% of the hospitalized HIV patients but only 43.2% of HIV negative subjects. In addition, hospitalized patients had a mean CD4 T lymphocyte count of 125 cells/µL and deaths attributed to sporotrichosis occurred 45 times more [83]. 

Clinical data from a systematic review showed the majority of HIV co-infected patients have cutaneous disease associated with involvement of other organs or systems. Their median CD4 T lymphocyte count was 97 cells/µL. In addition, unusual manifestations cannot be underestimated, with 17% of the cases presenting CNS involvement [84]. CNS disease has already been described in HIV-infected patients without clinical evidence of neurological symptoms [79,85,86]. Ten previous published cases with sufficient clinical details indicate poor prognosis for CNS involvement [85,86,87,88,89,90,91,92,93]. Thus, investigation of CNS disease in this specific population is strongly recommended [84] in order to provide early diagnosis and aggressive treatment. Most of these patients are males with a mean CD4 count of 101cells/µL. In all cases, skin lesions were present before or associated with the onset of meningeal symptoms. In addition, the concomitant involvement of lungs [85,89,92,93], mucosa [85,86,89], bone [86], kidney [93], testicles, epididymis, bone marrow, lymph nodes and pancreas [90] has also been described. 

In the majority of cases, patients present positive cerebrospinal fluid (CSF) cultures on admission or during the follow-up. Only in one case, lumbar puncture was sterile and *Sporothrix* sp. was observed in tissue sections [90]. Most importantly, however, biopsies of skin lesions yielded growth of *Sporothrix* spp. in almost all the previous cases [85,86,87,88,91,92,93], allowing the diagnosis to be made before CSF cultures results were available or became positive. Treatment of sporotrichosis meningoencephalitis is by far the most challenging aspect in the disease management and the poor therapeutic response observed in these few cases is remarkable. Except in one patient [93], amphotericin B formulations were the initial therapeutic choice, although a significant rate of recurrence or relapse of neurological symptoms was observed [85,86,92]. Nine of the patients died; the single patient who survived resolved the infection without sequels [92]. 

Other unusual manifestations associated with cutaneous lesions were endocarditis, mucosal involvement (ocular and nasal), uveitis, endophthalmitis, and pulmonary and osteoarticular involvement [12,24]. Isolated involvement of the lungs and sinus has also already been described [94,95,96,97]. 

The prevalence of HIV co-infection in patients with less severe forms is not yet well established, because HIV routine testing is not recommended for every patient with sporotrichosis. Usually, only those with severe manifestations or a suspected HIV infection result in a laboratory investigation, which would overestimate the incidence of severe clinical presentations and poor prognosis in this population. Nevertheless, Freitas et al. evaluated the prevalence of HIV co-infection in the Rio de Janeiro epidemic by testing stored blood samples of 850 patients with benign forms of sporotrichosis from 2000 to 2008, and found only one positive result [83]. Moreover, only a few cases of LC and FC forms have been described so far in HIV-infected patients with a mean CD4 T lymphocyte count of 513 cells/µL [12,83,98]. Overall, these data support the notion that HIV co-infection modifies the clinical presentation, severity and outcome of the patients with sporotrichosis, in accordance to their immune status and degree of immunosuppression [83]. 

## 5. Sporotrichosis Associated with IRIS

Only six cases of sporotrichosis associated with immune reconstitution inflammatory syndrome (IRIS) have been published to date. There are two cases of paradoxical sporotrichosis meningitis IRIS in patients who exhibited cutaneous lesion and were under itraconazole treatment before the onset of neurological disease. Both patients had confirmation of virologic response to antiretroviral therapy (ART). Despite treatment with amphotericin B at 4–8 weeks, the patients presented recurrence of neurological symptoms during follow-up with itraconazole and/or amphotericin B 2–3 times a week. *Sporothrix* sp. was isolated from CSF at some point [92]. 

These cases are controversial due to the difficulties in making a definite diagnosis of sporotrichosis IRIS when cultures remain positive, properly excluding other causes of clinical deterioration as therapeutic antifungal failure. Difficulties in defining IRIS still apply to other endemic mycoses such as paracoccidioidomycosis and cryptococcosis [99,100]. Although some debate persists, an apparent consensus for diagnosis of paradoxical IRIS associated with opportunistic mycoses is the worsening or appearance of new clinical and/or radiological manifestations consistent with an inflammatory process occurring during appropriate antifungal therapy with sterile cultures for the initial fungal pathogen within 12 months of ART initiation [100,101]. 

Another questionable report from Brazil described one patient with disseminated sporotrichosis who was already under treatment for two months with itraconazole when ART started. After six weeks, the patient experienced reactivation of old lesions and development of new cutaneous and mucosal lesions. However, cultures of skin biopsy were positive for *Sporothrix* sp. and the patient recovered well with increased doses of itraconazole combined with amphotericin B [92]. 

Lyra et al. described two cases of disseminated cutaneous sporotrichosis whose clinical presentations were more consistent with IRIS than progressive fungal infection or failure of treatment. Both patients started antifungal therapy shortly followed by ART. However, after four and five weeks, the patients exhibited paradoxical clinical worsening with recurrence of the lesion as well as development of new lesions along with systemic inflammatory symptoms such as fever and arthralgia. Subsequent mycological examination did not reveal fungal growth. The patients were treated with prednisone, resulting in rapid improvement of arthralgia and fever, followed by resolution of skin lesions [102]. 

Finally, one case described a patient who had no cutaneous findings before ART, but experienced unmasking of disseminated cutaneous sporotrichosis after five weeks. Cultures of lesion exudates were positive for *Sporothrix* sp. The patient’s cat had died of sporotrichosis one month before the patient started ART. He presented complete regression of the lesions after antifungal therapy [92]. 

### Case Presentation

**Figure 2:**
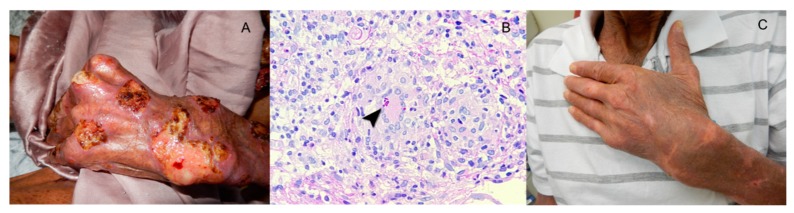
Ulcerated lesions in the hand and fist of a patient with human immunodeficiency virus (HIV) infection ad cutaneous disseminated sporotrichosis and immune reconstitution syndrome (**A**). A skin biopsy (**B**) depicted an exudative and granulomatous infiltrate with cigar shape and round yeast cells (arrow head), imbibed in multinucleate giant cells of the Langerhans type. Periodic Acid-Schiff stain × 400. The patient responded well to long course of continuous itraconazole intercalated with short courses of cotrimoxazole for secondary bacterial infection and prednisone for immune reconstitution inflammatory syndrome (IRIS) control (**C**).

A 59-year-old Brazilian man presented cachexia and disseminated and ulcerated skin lesions with one-year evolution (Figure 2A). Before his illness, he worked as an agriculturist, truck driver and a sewerage system cleaner in his town. During his last professional activity, he was continuously exposed to polluted water. Eight months earlier, the diagnosis of leprosy was made without any microbiological evidence and he was unsuccessfully treated with rifampin, dapsone and clofazimine. Six months ago, HIV infection was detected and lamivudine, tenofovir and efavirenz were added. At admission, he was depressed, febrile and complaining of pain. His body weight was 40 kg, and, besides the cutaneous clinical manifestations, there were no signs of internal organ involvement. The main laboratory findings included anemia with hemoglobin of 9.1 g/dL, leukocytosis (12,100 cell/μL) and protein chain reaction (PCR) of 11mg/L. HIV test was positive with CD4 cell count of 584 cells/mm^3^and viral load of 1558 copies/mL (log 3.1). A skin biopsy depicted a mixed exudative and granulomatous cellular infiltrate with a few round to elongated yeast cells (Figure 2B). The cultures of biopsy fragments yielded a dimorphic fungus phenotypically identified as *Sporothrix* sp., later identified by DNA sequence as *S. schenckii*. The anti-lepromatous therapy was stopped and the patient was treated with itraconazole, 400 mg per day, and cotrimoxazole 360mg/800mg per day for secondary bacterial infection. Because IRIS was suspected, prednisone at the daily dose of 20 mg per day was added and ART was changed to atazanavir/ritonavir due to probable drug-to-drug interactions between itraconazole and the previous antiretrovirals. He improved gradually and corticosteroid and cotrimoxazole were discontinued. After three months of therapy, itraconazole was reduced to 200 mg per day and discontinued after six months. The patient presented complete clinical and mycological responses. (Figure 2C). 

## 6. Comorbidities as Risk Factors for Sporotrichosis

### Diabetes Mellitus and Alcoholism

The main underlying disease reported in sporotrichosis outbreaks is diabetes mellitus, reaching up to 23% of the cases, followed by alcohol consumption, reaching from 5% to 8% [20,22,103]. Despite this observation, in hyperendemic areas of sporotrichosis, little is known about the contribution of these conditions to development of severe forms and there is limited understanding about the immunosuppressive mechanisms involved. To date, no large series or cohort studies described particularities of the clinical presentation in this population and most published reports of sporotrichosis in diabetes and alcoholism have focused on unusual manifestations, which are not necessarily the predominant forms observed in these populations.

Some of the first cases in alcoholic and diabetic patients date back to 1961 and 1970, respectively, and both patients presented with primary pulmonary sporotrichosis [104,105]. A recent systematic analysis of the literature addressing 86 cases of pulmonary sporotrichosis showed that diabetes mellitus was present in six patients and alcohol consumption in 34 cases. Most of these cases (75%) presented with primary pulmonary sporotrichosis. Of note, the cavitary pattern on radiology was the most common finding and 45% of the patients presented extrapulmonary involvement, the skin being the most affected site followed by joint involvement [4]. 

Putative differences in clinical presentation in diabetic and alcoholic patients occur. One presented disseminated cutaneous lesions and the other particularly severe/destructive localized lesions with a granulomatous aspect, denoting an enhanced pathogenicity in these localized cases. Nevertheless, all patients had a marked improvement with conventional antifungal treatment [106,107,108,109]. The most emblematic case of disseminated disease occurred in a diabetic and alcoholic patient who developed a fatal fungemia after 17 days of hospitalization [110]. The other cases corresponded to isolated monoarthritis, endophthalmitis and cutaneous disseminated sporotrichosis [111,112,113].

A retrospective study of 238 cases in the Peruvian highlands, where *S. schenckii* is hyperendemic, pointed to alcoholism and diabetes mellitus as significant underlying factors. The majority of patients had cutaneous or lymphocutaneous disease; only nine patients presented disseminated cutaneous disease and no cases of extracutaneous involvement were found [13]. In Brazil, clinical data from 178 patients with culture-positive sporotrichosis treated during the period of 1998–2001 showed that 80.9% of the cases presented LC or localized cutaneous forms. Systemic sporotrichosis was not diagnosed even in cases involving alcohol or diabetes as comorbidities, and in 29 patients (16.3%) with skin lesions at multiple locations, this was more likely due to repeated inoculations during persistent contact with sick animals [22]. 

In another Brazilian case of 24 patients with widespread cutaneous lesions, only two were associated with alcoholism and diabetes, and these conditions may have acted as an immunosuppressive factor favoring either the establishment of the infection and/or its dissemination. None of these presented a history of multiple exposures that could account for the widespread cutaneous lesions. In addition, none of the other patients showed any immunosuppressive condition and were found to be in good general condition, although fever and/or arthralgia were reported in 50% of the cases [103]. Furthermore, Rosa et al. described 304 patients where only four cases with cutaneous disseminated and extracutaneous forms were recognized, but data regarding their comorbidities were not provided [20].

## 7. Other Immunosuppressive Conditions

The literature reports several cases of cutaneous-disseminated and extra-cutaneous sporotrichosis in patients under immunosuppressive treatments for rheumatologic, autoimmune conditions, solid organ transplantation (SOT), hematologic cancers and primary immunodeficiencies. 

Osteo-articular or disseminated sporotrichosis misdiagnosed as rheumatoid arthritis, presumed inflammatory arthritis or sarcoidosis illustrates the role of iatrogenic immunosuppressive regimen in severity and complicated outcome [114,115,116]. Immunosuppressive therapy included steroids, azathioprine, tocilizumab, tacrolimus and cyclophosphamide—in one case, after almost one year of inappropriate therapy with several immunosuppressives (including prednisolone, tocilizumab, tacrolimus and cyclophosphamide), the patient experienced fungemia and died of respiratory insufficiency due to pulmonary sporotrichosis [114]. Two patients also had delayed diagnosis and progressed to disseminate disease albeit clinical improvement was achieved after antifungal therapy (amphotericin B or itraconazole) was started in parallel to lessening the iatrogenic immunosuppression [115,116]. 

A retrospective review of 19 cases of sporotrichosis diagnosed at a single service in the United States showed that seven patients were misdiagnosed initially and four received immunosuppressive agents for other diagnoses, such as polyarteritis nodosa, sarcoidosis, pyoderma gangrenosum and vasculitis [117]. One additional patient had received immunosuppressive therapy for a pre-existing polyarthropathy before the development of his cutaneous lesion. In contrast, none of the patients presented extracutaneous disease. The index case was diagnosed as pyoderma gangrenosum for disseminated leg ulcerated lesions and received immunosuppressive treatment with aziatropine, prednisone and cyclosporine with further worsening of the lesions. Treatment required debridement of necrotic tissue, plastic surgery and subsequent staged skin grafting, together with an 18-month course of 600mg/day itraconazole and cessation of immunosuppression. However, no data regarding the response to treatment of these 19 patients were provided. 

Similarly, cases of cutaneous disseminated and LC forms in immunosuppressive therapy with tacrolimus, anti-TNF-alpha and prednisone due to lupus nephritis, ankylosing spondylitis and sciatic pain, respectively, have been reported. All these patients had a good clinical response to antifungal therapy (potassium iodide or itraconazole) and discontinuation of immunosuppressive therapy [118,119,120].

The possible role of immunosuppressive drugs in atypical clinical presentation is reinforced by a systematic analysis of pulmonary sporotrichosis. In this study, of the 86 cases of pulmonary sporotrichosis included, 64 had primary pulmonary disease and 22 also had extra-pulmonary involvement. The only significant difference between the groups that could represent a risk factor for multifocal disease was the increased use of immunosuppressant drugs by the extra-pulmonary group [4]. 

Sporotrichosis in SOT recipients manifests as more severe disseminated forms than in immunocompetent hosts. Few exceptions respond well to antifungal treatment, being considered uncommon according to a prospective surveillance study of invasive fungal infections conducted in 15 SOT centers in United Sates, which did not identify any case of sporotrichosis [121]. However, in the Rio de Janeiro epidemic, sporotrichosis was retrospectively recognized in one subject, among 42 kidney transplant patients, with extracutaneous disease (LC and bone involvement) [122]. In addition, Caroti et al. followed 774 Italian kidney transplant patients for 18 years and subcutaneous nodules or cutaneous lesions were identified in seven [123]. One patient presented an erythematous papulonodular lesion with positive culture for *S. schenckii*. Despite treatment with fluconazole, seven years after renal transplantation, the patient developed acute osteomyelitis and gangrene in the left foot with ulcers. The patient was treated again with fluconazole together with interruption of the immunosuppressive agent mycophenolate mofetil, presenting gradual regression of the lesions. In India, during a period of two years, 40 renal transplants were performed and pulmonary sporotrichosis was diagnosed in one patient on triple drug immunosuppression [124]. Finally, there are three additional cases in kidney transplant recipients reported in the literature, one case of cutaneous disseminated and two of disseminated disease [23,125]. All patients were taking immunosuppressive agents and were successfully treated with antifungal therapy including amphotericin B deoxycholate, lipid amphotericin B formulations, fluconazole and itraconazole. One unusual case of urinary sporotrichosis after renal transplantation has also been described [25]. 

Sporotrichosis in SOT other than kidney transplantation is even more rare. Disseminated sporotrichosis with LC, articular and pulmonary involvement was described in a patient 10 years after liver transplantation still on immunosuppressive regimen (tacrolimus and prednisone) [23]. Despite antifungal treatment with itraconazole and reduction of immunosuppressant drugs, after 300 days of follow-up, the patient showed only partial improvement. Pulmonary sporotrichosis in a lung transplant recipient was also reported. On the second day of transplantation, while on induction of immunosuppression with high dose methylprednisolone, tacrolimus, and mycophenolate mofetil, he presented pulmonary diffuse patchy bilateral infiltrates and *S. schenckii* was isolated from bronchoalveolar lavage. Treatment with amphotericin B lipid formulation followed by itraconazole maintenance therapy was successful [126]. Rare cases of sporotrichosis in patients with hematologic cancer have also been described. One patient with multiple myeloma presented disseminated disease, successfully treated with amphotericin B [80]. Two Hodgkin’s disease patients, one with fatal meningeal sporotrichosis [127] and the other with a disseminated form refractory to potassium iodide, also responded to a long course of amphotericin B [80]. Two other cases reported patients with hairy cell leukemia, both with disseminated disease, one whose difficult and life-threatening course required liposomal amphotericin B followed by posaconazole (taken indefinitely), and the other exhibited a good responded to itraconazole [128,129].

Finally, primary immunodeficiency has been recognized as a risk factor for severe forms of the disease. A fatal case of disseminated form of sporotrichosis was described in one patient with primary idiopathic CD4 lymphocytopenia [130]. Another unusual case of *S. schenckii* cervical lymphadenitis was identified in a 33-month-old male with X-linked CGD that was successfully treated with surgical excision and voriconazole [131].

Overall, based mostly on published case reports, we suggest that patients on immunosuppressive regimen due to SOT, rheumatologic disease or other comorbidities are at higher risk of more severe clinical presentations of sporotrichosis. However, most cases presented a good outcome when provided with more prolonged and higher doses of antifungal treatment than used in immunocompetent hosts. In this scenario, the drug of choice should be guided by the severity of the disease, with initial therapy with amphotericin B being frequently required [114,116,117,125,126,127,128,130,132].

## 8. Laboratory Diagnosis

The most relevant diagnostic tool for patients with suspicion of sporotrichosis is isolation of etiologic agent from clinical specimens such as secretions, abscess aspirates and biopsied tissue fragments. In extracutaneous clinical forms, synovial fluid, blood, CSF and sputum should be cultivated. Fungal cultures may be obtained in standard media such as Sabouraud dextrose agar with antibiotics, Mycosel, blood agar and brain heart infusion media [133]. After 5 to 10 days, the yeast-like colonies may be observed at 37 °C incubation, although this time may be extended up to 30 days. For phenotypic identification, the micromorphology of mycelial forms must be seen after incubation at room temperature, although it cannot distinguish individual species. Species determination requires molecular methods for definitive identification [10,133].

**Figure 3:**
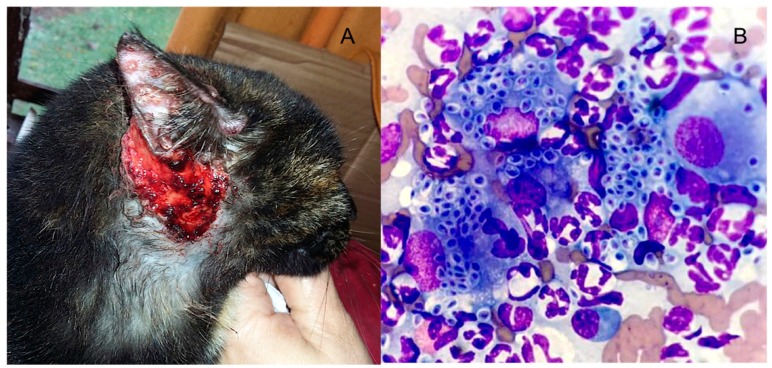
Ulcerated and papular vesicular lesions in the head and ear of a cat with proved *Sporothrix brasiliensis* infection (**A**). Cutaneous feline lesions are highly infective and harbor a great number of yeast cells of the fungus. Feline sporotrichosis can be easily diagnosed by secretion direct exam stained Giemsa × 1000 (**B**).

In contrast to immunocompetent patients, where direct mycologic or histopathologic exams show poor sensitivity, in immunocompromised hosts these methods may depict higher sensitivity, especially in AIDS patients with very low CD4 cell counts [83,84]. In severely immunocompromised AIDS patients, cutaneous and lymphatic lesions may depict a big fungal burden, similar to that observed in cats with *S. brasiliensis* infections [7,134] (Figure 3). Immunocompromised patients with cutaneous disseminated and extracutaneous clinical forms may depict *Sporothrix* spp. yeasts under immunofluorescence, Giemsa, Gram, Grocott-Gomori and periodic acid Schiff (PAS) stains. When observed, yeasts present round to oval and elongated “cigar shape” forms [2,10,135,136]. Non-microbiologic diagnostic tests such as immunoelectrophoresis, immunodiffusion, ELISA and DNA sequencing PCR methods are very important for typical and immune reactive forms but the only commercially available test for immunodiagnostic of sporotrichosis is the latex agglutination technique [10,137,138]. 

## 9. Treatment of Opportunistic Sporotrichosis

Therapy of patients with sporotrichosis associated with impaired host defenses does not differ from treatment modalities applied for immunocompetent individuals, except for inclusion of specific interventions for the underlying conditions leading to opportunistic disease. Although some experimental studies demonstrated different species might show variable in vitro sensitivity to systemic antifungal drugs, no clinical correlation in therapy of human sporotrichosis has been confirmed to date [133,138,139,140,141]. For patients with cutaneous or LC forms, itraconazole at the daily dose of 200–400 mg for 3–6 months is the therapy of choice. Exceptionally, if immunosuppression is maintained, a longer period of itraconazole therapy may be required [83,84]. Special attention is recommended for drug interaction between itraconazole and ART drugs such as efavirenz, ritonavir and darunavir [142]. If itraconazole is contraindicated due to intolerance, refractoriness or drug-to-drug interaction, 500 mg of terbinafine twice a day is the second option. Finally, for non-severe forms, super saturated potassium iodine solution, 40–50 drops three times per day, can be tried. Second generation triazoles as posaconazole and isavuconazole have not been evaluated yet. 

Although infection with *Sporothrix* spp. is rarely life threatening, all forms of sporotrichosis require some kind of treatment. Unlike HIV/AIDS patients, diabetic and alcoholic subjects do not seem to have a worse prognosis and, in general, all patients show a satisfactory response even if in some cases it is necessary to increase the drug dose or the hospitalization stay due to the comorbidity itself or the severity of the lesions [22]. 

Regardless of the presence of any comorbidity, in pulmonary, severe or life-threatening disease, amphotericin B should be the initial therapy until the patient shows a favorable response, moving on to itraconazole [140]. In parallel, the management of those risk factors, such as control of chronic alcohol intake and steroid or anti TNF discontinuation should be carried out.

An issue in HIV-patient management concerns the best time to introduce ART. While some authors recommend its initiation should be delayed in patients with CNS disease in order to avoid IRIS [84], currently there is no sufficient evidence to support this recommendation for secondary prophylaxis. Data from AIDS patients and tuberculosis or *Cryptococcus* meningitis suggest that patients should start antifungals before ART [143,144]. Therefore, similar to indications already described in the literature for other opportunistic mycoses, long-term suppressive therapy should be considered in patients with severe forms or CNS infection after at least 1 year of successful treatment and then discontinued in patients with CD4 cells counts ≥200 cells/µL and who have undetectable viral loads on ART for >6 months [140,145]. Because the risk of relapse of meningeal sporotrichosis is high, lifelong suppressive therapy seems prudent and recommended in these cases [140]. 

## 10. What Can Immunocompromised Patients with Sporotrichosis Teach Us?

Many issues in our understanding of sporotrichosis remain unresolved. First, even though it is expected, a report of specific exposure (i.e., sick cats, gardening, lumbering, farming, hunting, etc.) for contracting this implantation mycosis, such as an epidemiological link, was not evident in most cases compiled in this review, except for CTS. Thus, we hypothesize that the inhalatory route would be the most likely mode of infection. Moreover, in many cases retrieved in this review, sporotrichosis was not suspected initially, delaying diagnosis and appropriate treatment initiation, which might have contributed to a higher severity of the disease.

Second, a yet unknown number of the exposed individuals do not develop disease, suggesting the full list of predisposing factors is completely unknown. Although deficiencies of the host immune response are indisputably a critical factor, reports of systemic or severe extracutaneous disease in individuals without clinical or laboratorial evidence of immunodeficiency [79,80,81] indicates many factors should be considered in infection pathogenesis.

Not surprisingly, a frequent association between atypical forms of sporotrichosis and HIV/AIDS, transplantation, hematological malignancies and iatrogenic immunosuppression for rheumatologic conditions was detected. As seen for other endemic mycoses such as paracoccidioidomycosis, coccidioidomycosis and histoplasmosis, all these comorbidities, particularly HIV/AIDS, are high-risk factors for disseminated or atypical forms and can even change their natural history [146,147,148], mostly by promoting a defective T-cell immunity. This was suggested by the experimental works on nude mice reviewed here.

An interesting observation was identification of chronic alcohol abuse as a single predisposing factor to infection. The impact of chronic heavy alcohol consumption on the immune system is complex and time and dose dependent, typically resulting in a subclinical immunosuppression that becomes clinically significant only in the case of a secondary insult [149]. Innate immunity is affected, particularly by inhibiting cellular chemotaxis, phagocytosis (especially for alveolar macrophages) and production of growth factors [150]. Adaptive responses show severe compromise of T-cell function such as lymphopenia, increased cellular differentiation and activation, and reduced migration. The chronic activation of T-cell pool in alcoholic patients would alter its ability to expand and respond to pathogenic challenges or lead to their elimination through increased sensitivity to activation-induced cell death [149,151]. Although the higher risk of infections in alcoholic patients has been related mostly to bacterial (e.g., tuberculosis) and viral infections, our review points to chronic alcohol abuse as a risk factor of atypical, more severe, sporotrichosis. In addition, the higher susceptibility to infections by alcohol abuse may be in part related to behavioral changes that lead to enhanced exposure to pathogens.

Curiously, we only found two reports of primary immunodeficiencies patients with severe sporotrichosis (T CD4 lymphopenia and X-linked CGD). The latter corroborates the finding of increased susceptibility to sporotrichosis in the CGD mice model discussed earlier. Although studies of experimental sporotrichosis suggested that signaling via TLRs and other PRR would be crucial to recognition of the fungus and the mounting of effective immune responses, we did not find cases of OS associated with putative constitutive defects of these pathways. This may be due to the low frequency of these deficiencies in the general population compared with the immunosuppressed conditions aforementioned. We also did not find association between humoral immunodeficiencies and sporotrichosis, despite this subset of immunodeficiency being relatively more common. Thus, although antibodies are protective in some experimental models, they may not play a major role in human sporotrichosis.

Diabetes mellitus is an established risk factor for certain endemic mycoses (e.g., coccidioidomycosis, histoplasmosis, blastomycosis), but surprisingly not in others (e.g., paracoccidioidomycosis), and manifestations were more severe than in non-diabetic patients [152,153,154]. This was specially related to uncontrolled diabetes (chronic hyperglycemia). Decreased chemotaxis, phagocytic and killing activities of macrophages and neutrophils were described in uncontrolled diabetic patients [155]. However, as for chronic alcohol abuse, the (uncontrolled) diabetes mellitus induced alterations of both innate (neutrophils and macrophages) and adaptive immunity cells (mainly T cells) were related predominantly to enhanced susceptibility to tuberculosis [156,157,158]. Current evidence suggests underperforming innate immunity followed by a hyper-reactive T-cell-mediated immune response to *Mycobacterium tuberculosis* in patients with tuberculosis disease, but how these altered responses contribute to enhanced susceptibility or more adverse outcomes remains unclear. However, studies of latent tuberculosis individuals showed that diabetes leads to suboptimal induction of protective T-cell responses, thereby providing a possible mechanism for the increased susceptibility to active disease [158]. Conceivably, these alterations would apply to chronic granulomatous infections other than tuberculosis, such as those caused by fungal organisms. Thus, the observation of diabetes as the single underlying condition in an appreciable number of sporotrichosis patients should not be surprising. Further investigation is required to determine which of the immune dysfunctions presented by diabetic patients play a relevant role in the enhanced susceptibility to sporotrichosis.

## 11. Concluding Remarks and Future Perspectives

The data we gathered neither allow drawing definitive conclusions on several aspects of the opportunistic nature of sporotrichosis nor making a consensus on the management of these cases. However, they suggest that OS, when not life-threatening, frequently progresses to larger and/or deeper lesions that usually require higher doses of the antifungals and more prolonged courses of therapy. Therapy was generally started with amphotericin B formulations, which were moved to itraconazole after the initial improvement, as judged by the assisting clinician. In general, the patients responded well to treatment, even if slowly. The few fatalities were mainly accounted for by delayed onset of antifungal therapy or by use of immunosuppressors due to misdiagnosis (rheumatologic disease, sarcoidosis, etc.). Potassium iodide was seldom used (mostly in the early case reports), with poorer responses, being then replaced by amphotericin B. Notably, in addition to the skin, the most affected sites were bones, joints, lungs and CNS, with diagnosis based on histopathology/mycological examination plus cultures of specimens such as biopsies, synovial liquid, cerebrospinal fluid, and blood. Unfortunately, non-microbiologic tests, such as antibody/antigen detection, PCR and other molecular methods are not routinely applied for diagnosis. We thus urgently need standardized and commercially available diagnostic tools to discover the deep part of the sporotrichosis iceberg.
